# Predictive value of pre-procedural autoantibodies against M_2_-muscarinic acetylcholine receptor for recurrence of atrial fibrillation one year after radiofrequency catheter ablation

**DOI:** 10.1186/1479-5876-11-7

**Published:** 2013-01-07

**Authors:** Guiling Ma, Xinchun Yang, Jianjun Zhang, Lin Zhang

**Affiliations:** 1 Heart Center, Beijing Chao-Yang Hospital, Capital Medical University, 8 Gong-Ti South Road, Beijing 100020, China

**Keywords:** Atrial fibrillation, Radiofrequency catheter ablation, Muscarinic-2 acetylcholine receptor, Autoantibody, Recurrence

## Abstract

**Background:**

Increasing evidences have suggested that autoantibodies against muscarinic-2 acetylcholine receptor (anti-M_2_-R) may play an important role in the development of atrial fibrillation (AF). Predictive value of pre-procedural anti-M_2_-R for the recurrence of AF after radiofrequency catheter ablation is still unclear.

**Methods:**

Totally 76 AF patients with preserved left ventricular systolic function were prospectively enrolled and subjected to ablation after the detection of serum anti-M_2_-R by enzyme linked immunosorbent assay. These patients were given follow-up examination for one year after ablation. Risk estimation for the recurrence of AF was performed using the univariate and multivariate logistic regression.

**Results:**

In AF group, serum anti-M_2_-R was significantly higher than that in the control group in terms of frequency (40.8% versus 11.7%; *p* < 0.001) and titer (1:116 versus 1:29; *p* < 0.001). Compared with paroxysmal AF patients, persistent AF patients had higher frequency (57.6% versus 27.9%; *p* = 0.009) and titer (1:132 versus 1:94; *p* = 0.012) for autoantibodies. During one-year follow-up examination after ablation, the recurrence of AF was observed in 25 (32.9%) patients. Multivariate analysis showed that pre-procedural serum anti-M_2_-R was an independent predictor for the recurrence of AF at the time point of 12 months after ablation (odds ratio: 4.701; 95% confidence interval: 1.590-13.894; *p* = 0.005).

**Conclusions:**

In AF patients, the frequency and titer of serum anti-M_2_-R were significantly higher than those in the control group with sinus rhythm. Pre-procedural serum anti-M_2_-R was an independent predictor for the recurrence of AF one year after radiofrequency catheter ablation.

## Background

Atrial fibrillation (AF) is one of the most common arrhythmias in clinical setting, accounting for approximately one-third of hospitalizations for cardiac rhythm disturbances [1]. AF is associated with the reduced exercise capacity, degraded life quality, increased death rate, stroke and other thromboembolic events as well as left ventricular dysfunction [2]. To date, the underlying mechanism of AF is not fully understood, emerging evidences have indicated that autoimmunity may play an important role in the development of AF [3].

M_2_ muscarinic receptor belongs to the family of cardiac G-protein-coupled receptors. Circulating autoantibodies against the second extracellular loop of M_2_-muscarinic acetylcholine receptors (anti-M_2_-R) have been detected in a large number of cardiovascular diseases such as idiopathic dilated cardiomyopathy and chronic Chagas’s heart disease characterized by heart failure [4,5]. Anti-M_2_-R is also observed in a series of arrhythmic disorders including idiopathic atrial fibrillation and sinus node dysfunction and ventricular arrhythmias [6,7], suggesting that anti-M_2_-R is not only involved in heart failure but also in arrhythmia. Anti-M_2_-R has recently been reported as the strongest independent predictor for the development of AF in patients with idiopathic dilated cardiomyopathy and patients with Graves’ hyperthyroidism [6,8].

Over the past decades, radiofrequency catheter ablation (RFCA) has evolved rapidly and become one of the most effective methods for AF treatments so that it has been used in many major hospitals all over the world, although the recurrence rate of AF remains high [9]. Since AF is a heterogeneous disease, the identification of patients at high risk for the recurrence of AF by simple and objective parameters may be helpful in tailoring therapeutic strategies. Age, AF history, left ventricular dysfunction, left atrial dilation and increased plasma N-terminal pro-brain natriuretic peptide (NT-proBNP) level have been associated with an elevated recurrence rate of AF [10,11].

Therefore, the purposes of this study are to determine whether serum anti-M_2_-R can increase in patients with AF and preserved left ventricular systolic function, and to assess the predictive value of pre-procedural serum anti- M2-R for the recurrence of AF at one year after RFCA.

## Methods

### Study population

During the period from October 2009 to October 2010, a total of 76 patients with paroxysmal (n = 43) or persistent (n = 33) AF and preserved left ventricular systolic function (left ventricular ejection fraction, LVEF > 60%), who scheduled for RFCA in the heart center of Beijing Chao-Yang Hospital, were prospectively enrolled. The definition and classification was described in the guidelines for AF [2]. Exclusion criteria were intra-cardiac thrombi especially in left atrium and left atrial appendage, LVEF < 50%, renal insufficiency (serum creatinine ≥ 176.8 μmol/L), a history of congenital heart disease, rheumatic valvular heart disease, idiopathic cardiomyopathy, hyperthyroidism or other autoimmune diseases. Patients with contraindication to anticoagulation or allergy to iodide were also excluded from this study. Meanwhile, totally 77 patients presented for primary hypertension or coronary artery disease or just for medical checkup during the same period with sinus rhythm, LVEF > 60%, and age within 5 years in comparison with AF patients were selected as the control group. Baseline clinical characteristics were collected and plasma NT-proBNP level was measured in all subjects. Trans-thoracic echocardiography was used to evaluate left atrial diameter and left ventricular function. Trans-esophageal echocardiography should be performed to confirm that there was no thrombus in the left atrium or left atrial appendage. The study was complied with the Declaration of Helsinki and was approved by the Ethics Committee of Beijing Chao-Yang Hospital. All subjects were provided the written informed consent before study.

### Study protocol

Treatment with antiarrhythmic drugs of all patients was discontinued for three days before the procedure; amiodarone was withdrawn at least 6 weeks before hospitalization. Oral anticoagulation drugs were replaced by subcutaneous low molecular weight heparin for at least 24 hours. Blood samples were collected before RFCA and centrifuged at 3000 rpm for 10 minutes. The anti-M_2_-R was measured with enzyme linked immunosorbent assay (ELISA), as described by Fu [4].

All 76 patients with AF were subjected to circumferential ablation of pulmonary vein (CAPV) by applying radio-frequency energy at the conditions with environmental temperature of 43°C and limited power of 43 watts. Guided by 3D electro-anatomical mapping systems such as CARTO, continuous circumferential lesions that encircled the right and left pulmonary vein ostia were performed during sinus rhythm or AF. The end point of CAPV was the elimination or dissociation of pulmonary venous potentials throughout the ostial circumference. If AF persisted or recurred during paced rhythm after CAPV, additional substrate modification, for example, an additional linear ablation of the left atrium “roof” line, or “mitral isthmus” line may be required and the end point of linear ablation was documented as bi-directional conduction block around linear ablation lesions.

### Follow-up examination

An echocardiogram was performed immediately after the procedure to exclude the presence of pericardial effusion. All patients were subjected to hospital electrocardiogram telemetry monitoring for 72 hours. After hospital discharge, all of the enrolled patients with AF were scheduled for repeated visits in an outpatient clinic at 1, 2, 3, 6 and 12 months after the ablation. At each visit, patients were provided with cardiological examination and intensive questioning for arrhythmia-related symptoms (palpations, chest discomfort, fatigue and dizziness) at the heart center and a 12-lead electrocardiogram was also performed. Moreover, a 24-hour Holter recording was conducted in 3, 6 and 12 months for all of the patients. The recurrence of AF was defined as any symptomatic or detected episodes of AF, atrial flutter and atrial tachycardia longer than 30 seconds beyond the blanking period of three months without anti-arrhythmic drugs [9].

### Statistical methods

Continuous data were expressed as Mean ± SD and categorical data were expressed as percentages. For group comparisons, the Student’s *t* test for continuous variables and the chi-square test or Fisher’s exact test for categorical variables were used. Association between serum anti-M_2_-R level and the echocardio-graphic or clinical indexes was evaluated by Spearman’s correlation. Risk estimation was performed using univariate and multivariate logistic regression model with the presence of recurrence of AF as dependent variable. All tests were 2-tailed, and a significant difference was considered at the *p* < 0.05. Statistical analysis was performed using the SPSS 16.0 statistical software package (SPSS, Inc., Chicago, Illinois).

## Results

### Patient profiles

The clinical profiles of the study population were summarized in Table 1. Compared to patients with sinus rhythm, AF patients had larger left atrial diameter (39.86 ± 5.35 mm vs. 35.33 ± 5.86 mm; *p* < 0.001) and higher NT-proBNP level (213.85 ± 85.76 pg/mL versus 113.42 ± 31.15 pg/mL; *p* < 0.001). Demographic characteristics and underlying diseases were similar between two groups. Echocardiographic data including left ventricular end-diastolic dimension (LVEDD), left ventricular end-systolic dimension (LVESD) and LVEF did not reveal a significant difference between two groups.

**Table 1:** Clinical profiles of the study population

**Variables**	**Sinus rhythm (n = 77)**	**Atrial fibrillation (n = 76)**	***p *** **value**
Age (years)	65.68 ± 9.24	65.34 ± 8.93	0.817
Gender (male)	36 (46.8%)	39 (51.3%)	0.572
Hypertension (%)	36 (36.8%)	37 (48.7%)	0.811
Diabetes mellitus (%)	14 (18.2%)	22 (28.9%)	0.117
Coronary artery disease (%)	8 (10.4%)	9(11.8%)	0.775
**Left atrial diameter (mm)**	**35.33 ± 5.86**	**39.86 ± 5.35**	**< 0.001**
Left ventricular end-diastolic diameter (mm)	45.34 ± 5.10	46.42 ± 3.92	0.270
Left ventricular end-systolic diameter (mm)	27.55 ± 3.32	28.04 ± 3.26	0.359
Left ventricular ejection fraction (%)	69.13 ± 4.97	69.17 ± 5.98	0.979
**NT-proBNP (pg/ml)**	**113.42 ± 30.15**	**213.85 ± 85.76**	**< 0.001**

Data were expressed as numbers (%), Mean ± SD.

Abbreviations: NT-proBNP = N-terminal pro-brain natriuretic peptide.

### Autoantibody screening

**Figure 1:**
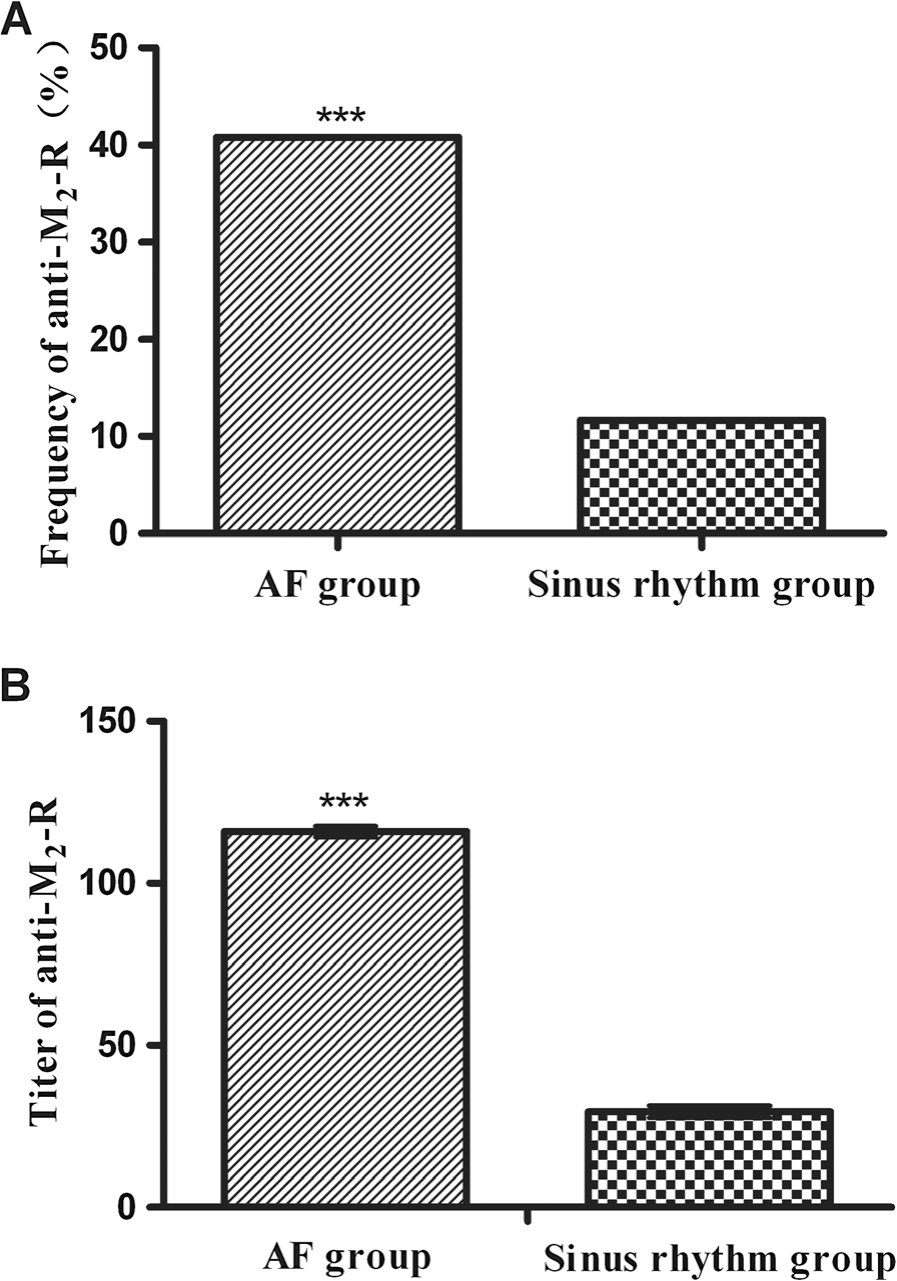
**Difference in anti-M**_**2**_**-R between atrial fibrillation and sinus rhythm patients.** The frequency of anti-M_2_-R in AF patients was 40.8%, which was significantly higher than that of 11.7% in patients with sinus rhythm (**A**) (P < 0.001). The geometric mean titer in AF patients was also significantly higher than that in patients with sinus rhythm (**B**) (1:116 versus 1:29; P < 0.001).

Anti-M_2_-R was detected in 40.8% (31 of 76) of AF patients, whereas that of 11.7% (9 of 77) in patients with sinus rhythm (*p* < 0.001). Moreover, in positive cases, autoantibody titer (geometric mean) in AF patients was significantly higher than that in patients with sinus rhythm (1:116 versus 1:29; *p* < 0.001), as shown in Figure 1. Subgroup analysis showed a significant difference in the frequency of anti-M_2_-R (57.6% versus 27.9%; *p* = 0.009) and autoantibody titer (1:132 versus 1:94; *p* = 0.012) between persistent and paroxysmal AF patients.

### Ablation outcome

The end points of pulmonary vein disconnection or elimination in all 76 patients with AF after CAPV were achieved. Totally, 17 of 33 patients with persistent AF (51.5%) and 9 of 43 patients with paroxysmal AF (20.9%) were subjected to additional linear ablation. As for the population in the present study, no patient was subjected to repeated ablation.

### Follow-up outcome

During one-year follow-up examination after the ablation, the recurrence of AF was observed in 25 (32.9%) patients. The baseline characteristics of two groups with and without recurrent AF were listed in Table 2. No statistical difference in age, gender, body mass index, smoking, comorbidity (hypertension, diabetes mellitus and coronary artery disease), medications and renal function between both groups was observed. The echocardiographic data including LVEDD, LVESD and LVEF were also similar between both groups at baseline. Furthermore, there is no significant difference in the ablation protocol between two groups as well.

**Table 2:** Characteristics of patients with and without atrial fibrillation recurrence after ablation

**Variables**	**No recurrence (n = 51)**	**AF recurrence (n = 25)**	***p*** **value**
Age (years)	65 ± 9	66 ± 8	0.728
Gender (male)	26 (51.0%)	13 (52.0%)	0.933
Body mass index (kg/m^2^)	25.74 ± 3.46	26.13 ± 3.65	0.710
Smoking (%)	21 (41.2%)	7 (28.0%)	0.263
**Persistent atrial fibrillation (%)**	**14 (27.5%)**	**19 (76.0%)**	**< 0.001**
Duration of atrial fibrillation (months)	24 ± 5	60 ± 15	0.090
Comorbidity			
Hypertension (%)	26 (51.0%)	11 (44.0%)	0.567
Diabetes mellitus (%)	13 (25.5%)	9 (36.0%)	0.343
Coronary artery disease (%)	5 (9.8%)	4 (16.0%)	0.432
Medications			
Statins	22 (43.1%)	12 (48.0%)	0.689
ACEI/ARB	40 (78.4%)	18 (72.0%)	0.536
Renal function			
Blood urea nitrogen (mmol/L)	5.67 ± 1.60	5.43 ± 1.87	0.630
Creatinine (μmol/L)	77.58 ± 16.81	79.75 ± 8.85	0.526
Creatinine clearance (mL/min)	83.12 ± 28.23	79.06 ± 22.86	0.616
Echocardiographic data			
** Left atrial diameter (mm)**	**39.0 ± 3.5**	**44.9 ± 2.1**	**< 0.001**
Left ventricular end-diastolic diameter (mm)	46.3 ± 3.9	46.9 ± 4.2	0.593
Left ventricular end-systolic diameter (mm)	28.0 ± 3.1	27.5 ± 2.1	0.411
Left ventricular ejection fraction (%)	69.0 ± 5.8	70.0 ± 3.5	0.354
**NT-proBNP (pg/ml)**	**204.50** ± **56.35**	**347.80** ± **35.80**	**< 0.001**
Ablation protocol (persistent AF)	n=14	n=19	
CAPV+additional linear ablation	8 (57.1%)	9 (47.4%)	0.728
CAPV+LA "roof" line	8 (57.1%)	9 (47.4%)	0.728
CAPV+LA "mitral isthmus" line	8 (57.1%)	9 (47.4%)	0.728
Ablation protocol (paroxysmal AF)	n=37	n=6	
CAPV+additional linear ablation	7 (18.9%)	2 (33.3%)	0.589
CAPV+LA "roof" line	7 (18.9%)	2 (33.3%)	0.589
CAPV+LA "mitral isthmus" line	5 (13.5%)	0	-

Data were expressed as numbers (%), Mean ± SD.

Abbreviations: ACEI = angiotensin-converting enzyme inhibitor, ARB = angiotensin II receptor blocker, NT-proBNP = N-terminal pro-brain natriuretic peptide,CAPV=circumferential ablation of pulmonary vein, LA=left atrium.

Patients with the recurrence of AF, however, had more persistent AF (76.0% versus. 27.5%; *p* < 0.001), larger left atrial diameter (44.9 ± 2.1mm versus 39.0 ± 3.5mm; *p* < 0.001), higher plasma NT-proBNP level (347.80 ± 35.80 pg/mL versus 204.50 ± 56.35 pg/mL; *p* = 0.012) when compared to patients without AF recurrence. No significant difference in AF duration was observed in recurrent patients when compared with patients without AF recurrence (60 ± 15 months versus 24 ± 5 months; *p* = 0.090).

**Figure 2:**
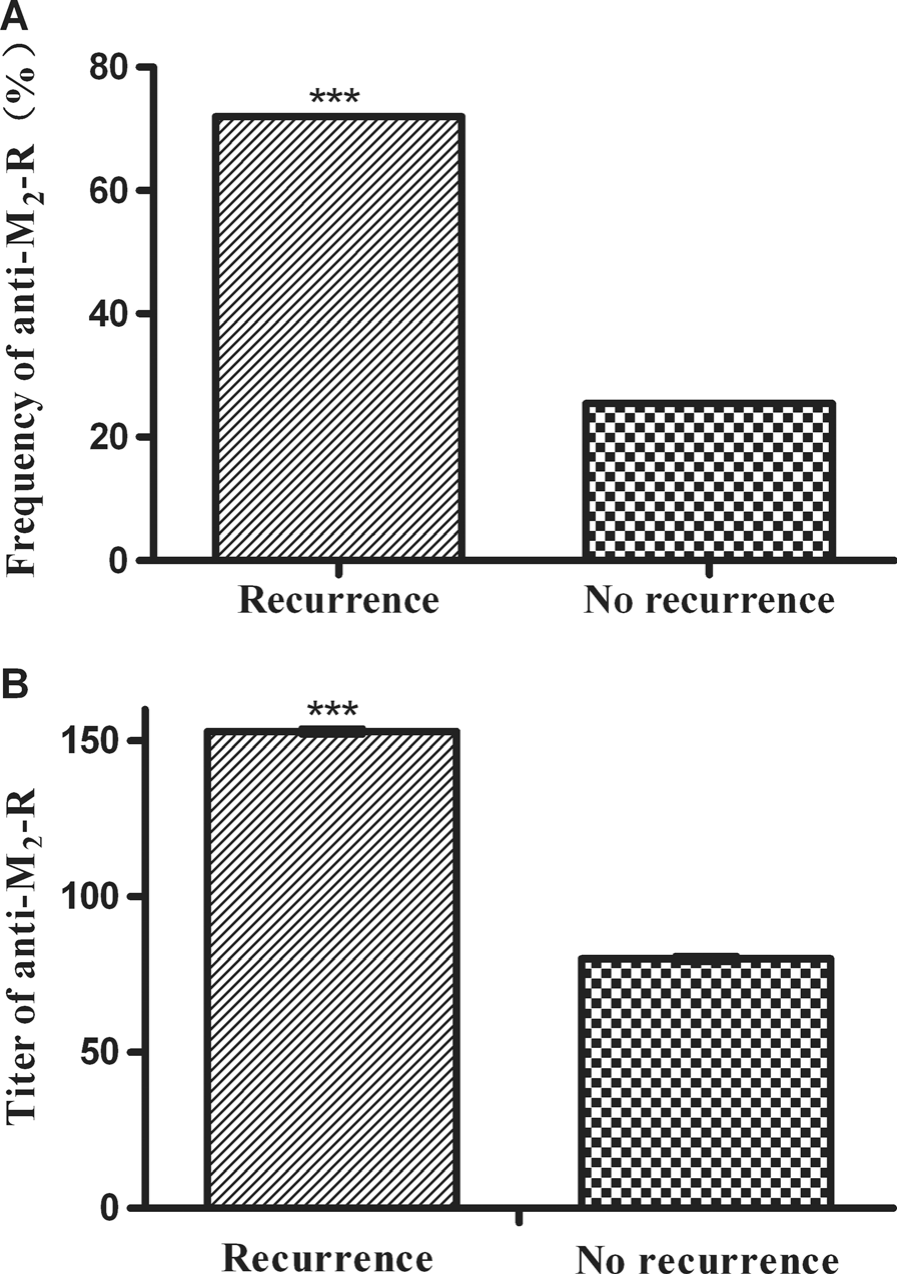
**Difference in anti-M**_**2**_**-R between patients with and without recurrence.** The frequency of anti-M_2_-R in the patients with recurrent AF was significantly higher than that without recurrence (**A**) (72.0% versus 25.5%; P < 0.001). The geometric mean titer of anti-M_2_-R in the recurrent patients was also higher than that in the patients without recurrence (**B**) (1:153 versus 1:80; P = 0.002).

As for the autoantibodies, not only the frequency of anti-M_2_-R (72.0% versus 25.5%; *p* < 0.001), but also the titer of anti-M_2_-R (1:153 versus 1:80; *p* = 0.002) revealed a significant difference between the patients with and without AF recurrence, as shown in Figure 2.

### Determinant of serum anti-M_2_-R level

The titer of serum anti-M_2_-R was highly correlated with left atrial diameter (R = 0.490; *p* < 0.001) and plasma NT-proBNP level (R= 0.502; *p* < 0.001). However, age, body mass index, renal function, AF duration and LVEDD, LVESD as well as LVEF were not correlated with serum anti-M_2_-R level (*p* > 0.05).

### Univariate and multivariate analysis of AF recurrence

Univariate analysis for AF recurrence during one-year follow-up period was shown in Table 3. The variables such as left atrial diameter, pre-procedural plasma NT-proBNP level and serum anti-M_2_-R level (both frequency and titer) were significantly associated with AF recurrence at the time point of one year after RFCA including persistent AF. In the multivariate logistic regression, adjusted by persistent AF, left atrial diameter and pre-procedural plasma NT-proBNP level, not only pre-procedural titer of serum anti-M_2_-R (odds ratio: 2.339; 95% confidence interval: 1.509-3.626; *p* < 0.001) but also the presence of anti-M_2_-R (odds ratio: 4.701; 95% confidence interval: 1.590-13.894; *p* = 0.005) was an independent predictor for the recurrence of AF at one year after RFCA.

**Table 3:** Univariate and multivariate analysis for atrial fibrillation recurrence after ablation

**Variables**	**Univariate analysis**			**Multivariate analysis**		
	**OR**	**95% CI**	***p *** **value**	**OR**	**95% CI**	***p *** **value**
Age (years)	1.009	0.951-1.071	0.758			
Gender (male)	0.935	0.337-2.579	0.897			
**Persistent AF**	**3.794**	**1.350-10.659**	**0.011**			
Duration of AF (months)	1.005	0.998-1.012	0.189			
**Left atrial diameter (mm)**	**1.108**	**1.002-1.225**	**0.045**			
LVEF (%)	1.047	0.961-1.141	0.289			
**NT-proBNP**	**1.001**	**1.000-1.002**	**0.007**			
Anti-M_2_-R						
**Titer**	**4.252**	**1.913-6.591**	**<0.001**	**2.339**	**1.509-3.626**	**<0.001**
**Frequency**	**5.500**	**1.880-16.091**	**0.002**	**4.701**	**1.590-13.894**	**0.005**

Abbreviations: OR=odds ratio, CI=confidence interval, AF=atrial fibrillation, LVEF=left ventricular ejection fraction, NT-proBNP = N-terminal pro-brain natriuretic peptide, anti-M_2_-R = autoantibody against muscarinic-2 acetylcholine receptor.

## Discussion

### Major findings

By using a synthetic peptide corresponding to the sequence of the second extracellular loop in human M_2_ receptor, we have detected anti-M_2_-R with ELISA and demonstrated that the frequency and titer of serum anti-M_2_-R in patients with AF and preserved left ventricular systolic function were significantly higher than those in the patients with sinus rhythm. Both frequency and titer of serum anti-M_2_-R were associated with the left atrial diameter and plasma NT-proBNP level. The pre-procedural level of serum anti-M_2_-R was an independent predictor of AF recurrence at one year after RFCA. This study suggested the serum anti-M_2_-R could be used as an integrating marker for various risk factors of AF, and as an incremental predictive marker for the recurrence of AF after RFCA. To date, this is the first report in which pre-procedural serum anti-M_2_-R level was used to predict the recurrence of AF at one year after RFCA.

### Detection of serum anti-M_2_-R

Many studies have shown that circulating anti-M_2_-R can be frequently identified in different cardiovascular conditions such as idiopathic dilated cardiomyopathy, chronic Chagas’s heart disease, and various arrhythmic disorders, as well as in healthy individuals [11]. We have found that the frequency of anti-M_2_-R in patients with AF and preserved left ventricular systolic function was 40.8%, which was moderately higher than 23.1% in idiopathic AF reported by Baba et al. [6]. Furthermore, the titer of serum anti-M_2_-R is significantly higher in patients with AF than that in the patients with sinus rhythm.

### Role of serum anti-M_2_-R in the development of AF

The underlying mechanism of AF is still not fully understood. Over the past decades, multiple-circuit reentry and focal electrical triggers are the major conceptual models of AF [9]. AF is associated with the reduced potential duration and effective refractory period, which is mediated by the activation of the acetylcholine-gated potassium channel I_K-Ach_[12]. These electrophysiological changes (a process called “electrical remodeling”) promote the initiation and maintenance of AF [13]. Besides, many studies have also found that AF patients often have a larger left atrial diameter and greater atrial fibrosis (a process called “structure remodeling”), which partly attributes to the induction and persistence of AF through decreasing the atrial conduction velocity and prolonging intra-atrial activation time [14]. Autonomic nervous system plays an important role in the development of AF. Vagal stimulation shortens atrial action potential duration and effective refractory period, and increases the dispersion of atrial effective refractory period, which can create an arrhythmogenic substrate for the initiation and perpetuation of AF [15]. Serum anti-M_2_-R can specifically recognize and bind to amino acids 169–193 of the second extracellular loop in the M_2_-muscarinic acetylcholine receptor. Functional studies have demonstrated that these autoantibodies are not only able to bind to target receptors in the myocardium, but also to induce receptor-mediated biological responses as partial agonists [15]. Stavrakis S et al. [8] have found that in patients with Graves’ hyperthyroidism, the presence of anti-M_2_-R can facilitate shortening of action potential duration and local autonomic nerve stimulation-induced firing in pulmonary veins. By studying the effect of anti-M_2_-R on rabbit atria *in vivo*, Hong CM et al. [16] have demonstrated that anti-M_2_-R can induce atrial electrophysiology remodeling, atrial fibrosis and exert a tonic activation of M_2_ receptor signal transduction. These results suggest the presence of anti-M_2_-R may participate in the induction and perpetuation of AF.

Our present study find both the frequency and the titer of serum anti-M_2_-R are positively correlated with left atrial diameter. Moreover, AF patients with anti-M_2_-R are more prone to AF recurrence after ablation than the patients with autoantibody deficiency, which further confirmed the hypothesis that anti-M_2_-R may play an important role in the initiation and persistence of AF.

### Predictive value of anti-M_2_-R for the recurrence of AF after ablation

Since AF is a heterogeneous disease, the identification of patients at high risk for the recurrence of AF using simple and objective parameters may be helpful in tailoring the therapeutic strategies. According to previous studies, old age, long AF history, persistent AF, left ventricular dysfunction (systolic and/or diastolic dysfunction), left atrial dilation and increased plasma NT-proBNP level have been associated with an elevated recurrence rate of AF [10,11].

In the present study, the AF patients with preserved left ventricular systolic function have been used as subjects to conduct univariate analysis and the results have demonstrated that the variables such as enlarged left atrial diameter and increased pre-procedural plasma NT-proBNP level are significantly associated with the recurrence of AF at one year after RFCA including persistent AF, which is in good agreement with several previous studies [10,11,17]. Besides, both the frequency and the geometric mean titer of anti-M_2_-R reveal significant difference between the patients with AF recurrence and the patients with sinus rhythm restoration. Multivariate logistic regression analysis indicated that pre-procedural titer and frequency of serum anti-M_2_-R were independent predictors for recurrence of AF at one year after RFCA.

### Study limitations

Several limitations of our study must be acknowledged. We did not measure the levels of anti-M_2_-R after ablation, consequently being not able to demonstrate the superiority of baseline anti-M_2_-R as a predictor over a change in its levels. In addition, we did not perform echocardiography at one year after ablation, which could have been used to assess the effect of successful ablation on the left atrial size. The subgroup analysis, although it is of interest, is limited by the relatively small number of study population. Finally, in this study, the AF recurrence might have been underestimated because only intermittent 24 h Holter recording and interviews could results in mis-recording of asymptomatic AF recurrence during the clinical follow-up.

## Conclusions

In patients with AF and preserved left ventricular systolic function, the frequency and titer of serum anti-M_2_-R are higher than those in the patients with sinus rhythm. Meanwhile, serum anti-M_2_-R level is associated the left atrial diameters and plasma NT-proBNP level. The pre-procedural level of anti-M_2_-R is an independent predictor for the recurrence of AF at one year after ablation.

## Competing interests

The authors declare that they have no competing interests.

## Authors’ contributions

CHZ, ZYZ, WMZ, and GL carried out the case collection and follow-up examination. CHZ and GLM carried out the immunoassay. XCY participated in the design of the study and performed the statistical analysis. LZ and JJZ contributed the whole study and participated in the design and coordination of this project as well as manuscript writing. All authors reviewed and approved the final manuscript.
